# Reduction and Removal of Chromium VI in Water by Powdered Activated Carbon

**DOI:** 10.3390/ma11020269

**Published:** 2018-02-09

**Authors:** Yanan Chen, Dong An, Sainan Sun, Jiayi Gao, Linping Qian

**Affiliations:** 1 Department of Environmental Science & Engineering, Fudan University, 220 Handan Road, Shanghai 200433, China; 16210740021@fudan.edu.cn (Y.C.); 17110740033@fudan.edu.cn (S.S.); 15210740022@fudan.edu.cn (J.G.); 2 Shanghai Institute of Pollution Control and Ecological Security, Shanghai 200092, China; 3 Department of Chemistry, Fudan University, 220 Handan Road, Shanghai 200433, China; lpqian@fudan.edu.cn

**Keywords:** chromium, activated carbon, wastewater, adsorption

## Abstract

Cr adsorption on wood-based powdered activated carbon (WPAC) was characterized by scanning electron microscopy coupled with energy dispersive spectroscopy (SEM-EDS), Raman spectroscopy, and X-ray photoelectron spectroscopy (XPS). The highest Cr(VI) adsorption (40.04%) was obtained under acidic conditions (pH 3), whereas Cr removal at pH 10 was only 0.34%. The mechanism of Cr(VI) removal from aqueous solutions by WPAC was based on the reduction of Cr(VI) to Cr(III) with the concomitant oxidation of C-H and C-OH to C-OH and C=O, respectively, on the surface of WPAC, followed by Cr(III) adsorption. Raman spectroscopy revealed a change in the WPAC structure in terms of the *D*/*G* band intensity ratio after Cr(VI) adsorption. SEM-EDS analysis showed that the oxygen/carbon ratio on the WPAC surface increased from 9.85% to 17.74%. This result was confirmed by XPS measurements, which showed that 78.8% of Cr adsorbed on the WPAC surface was in the trivalent state. The amount of oxygen-containing functional groups on the surface increased due to the oxidation of graphitic carbons to C-OH and C=O groups.

## 1. Introduction

Chromium (Cr) is a common heavy metal pollutant in water, where it mainly exists in two stable oxidation states, i.e., hexavalent chromium (Cr(VI)) and trivalent chromium (Cr(III)) [1]. Toxicity studies have shown that Cr can enter the human body through the respiratory tract and skin, and has significant carcinogenic and mutagenic effects. Cr(VI) is about 100 times more toxic than Cr(III) because of its higher solubility and easy absorption and accumulation in kidneys, stomach, and liver [2]. Cr-containing compounds are widely used in various industries such as leather, electroplating, textile dyeing, and metal fabrication and finishing. Cr-containing wastewaters are one of the major pollutants of the environment [3]. According to the World Health Organization, the permissible limit of Cr(VI) in drinking water is 0.05 mg/L [4], whereas a limit of 0.1 mg/L was recommended by the United States Environmental Protection Agency [5].

Various methods have been developed for the removal of Cr(VI) from wastewater such as filtration membrane, ion exchange, reverse osmosis, precipitation, electrochemical treatment, solvent extraction, and adsorption/biosorption [6,7,8,9,10,11]. Recently, the Fe(II) reduction of Cr(VI) as an innovative available method has been proved to be very efficient and under appropriate combination of technologies, the amount of reagent (Fe-II) can be reduced to nearly stoichiometric ratios [12]. Among these, adsorption has been extensively applied due to its great advantages such as easy operation, high efficiency, and low cost. In particular, activated carbon is an excellent adsorbent with good physical and chemical properties including large surface area, high porosity, and strong adsorption capacity [13,14]. Also, recent developments including Cr(VI) and arsenic removal by activated carbon modified by irons revealed the importance and specification of the use of activated carbon [15,16].

The mechanism of Cr(VI) removal by activated carbon has been investigated in order to improve the adsorption efficiency. The electrostatic attraction and complexation with the functional groups on the activated carbon surface were found to be responsible for the removal of Cr(VI) [17,18,19]. Moreover, Gong et al. [20] and Ihsanullah et al. [21] proposed that Cr(VI) is reduced to the less toxic Cr(III) by adsorption onto activated carbon under acidic conditions. However, in these studies, only the adsorption experimental results were discussed, and to the best of our knowledge, the mechanism has not yet been investigated using modern spectroscopic techniques such as scanning electron microscopy coupled with energy dispersive spectroscopy (SEM-EDS), X-ray photoelectron spectroscopy (XPS), and Raman spectroscopy. Such analyses would provide further insights into Cr adsorption to help improve the removal rates.

Park et al. [22], Huang et al. [14], and Wu et al. [9] examined the Cr(VI) adsorption mechanism by XPS to identify the elements and the valence of the metal ions on the surface of activated carbon. Ozdemir et al. [23] and Dubey et al. [24] determined the atomic concentration of the adsorbed species by SEM-EDS, which is commonly used to characterize the morphology and the composition of the solid material. Raman spectroscopy is a powerful tool for characterizing carbon materials because of the wide variety of spectral shapes corresponding to the forms of carbon, revealing fine structural information [25]. Moreover, Xu et al. [26] used Raman spectroscopy to evaluate the degree of surface structural change of activated carbon fabrics treated with Cr(VI). Yet, to our knowledge, a combination of these methods has not been used to confirm the mechanism of Cr(VI) removal.

The main purpose of this study was to investigate the mechanism of the reaction between the toxic Cr(VI) and activated carbon by batch adsorption experiments. The mechanism of Cr(VI) removal from aqueous solutions was characterized by SEM-EDS, Raman spectroscopy, and XPS analyses.

## 2. Materials and Methods

### 2.1. Powdered Activated Carbon

Wood-based powdered activated carbon (WPAC, 200 mesh) was purchased from Calgon Carbon Corp. (Pittsburgh, PA, USA). Virgin WPAC was characterized by the following parameters: an iodine number of >900 mg/g, determined by the iodometric method; a methylene blue number of ≥120 mg/g, measured from the absorbance change of methylene blue solutions; and a Brunauer–Emmett–Teller (BET) surface area of >1000 m^2^/g, measured according to the ASTM D6556-10 standard method [27]. The details are provided in the ASTM D4607-94 standard method [28].

### 2.2. Determinations

The pH values were determined by a pH meter (Mettler Toledo Instruments Co., Ltd., Greifensee, Switzerland). Sodium hydroxide and hydrochloric acid were purchased from Sinopharm Chemical Reagent Co., Ltd. (Shanghai, China).

A stock solution of 1000 mg/L Cr(VI) was obtained from Guobiao Testing and Certification Co., Ltd. (Beijing, China) derived from potassium dichromate.

The concentration of Cr(VI) was measured using a ChromaVer^®^ 3 Reagent Powder Pillow to produce a colorimetric response, which was measured using a HACH DR6000 spectrophotometer (HACH, Loveland, CO, USA) at 540 nm.

All experiments were conducted using deionized water (Milli-Q, Millipore, Billerica, MA, USA). 

### 2.3. Batch Tests

Batch Cr(VI) adsorption studies were performed by mixing 20 mg WPAC with 50 mL of an 80 mg/L Cr(VI) solution in a 250-mL Erlenmeyer flask. The flask was sealed and shaken in a thermostatic shaker (Shanghai Tianhe Automation Instrumentation Co., Ltd., Shanghai, China) at a speed of 180 rpm at 25 °C in the dark. After equilibration for 24 h, WPAC was filtered through a 0.45-μm membrane, and the residual concentration of Cr(VI) was measured. The experimental conditions were selected to be optimal according to batch preliminary experiments. All tests were performed in duplicate with an error of <5%. The initial solution pH value was tested around 7, which indicated neutral conditions. In order to investigate the influence of different pH values on adsorption capacity, the initial pH of the experimental solutions was adjusted to the desired value (pH 3, 10) by adding 0.1 M H_2_SO_4_ or 0.1 M NaOH. The samples before and after Cr(VI) adsorption at pH 3, 7, and 10 were dried for the following SEM-EDS, Raman, and XPS analysis.

The percent removal of Cr(VI) and equilibrium adsorption capacity (*q_e_*) of Cr(VI) were calculated using Formulas (1) and (2), respectively:

(1) $$\mathrm{Cr}(\mathrm{VI})\;\mathrm{removal}\;\mathrm{rate}\;(\%)=\frac{C_{0}-C_{e}}{C_{0}}\times 100$$

(2) $$q_{e}=\left[\frac{\left(C_{0}-C_{e}\right)}{m}\right]\times V$$

where *C*_0_ (mg/L) and *C_e_* (mg/L) are the initial and equilibrium concentrations of Cr(VI), respectively; *V* is the volume of the solution; and *m* (g) is the weight of the WPAC.

### 2.4. Parameters of SEM-EDS, Raman Spectroscopy, and XPS

SEM analysis was carried out using a PHILIPS XL 30 microscope (Philips Company, Amsterdam, The Netherlands) equipped with an energy dispersive analytical system operating at an accelerating voltage of 20 kV.

Raman spectra were recorded in the range of 300–3000 cm^−1^ by using an XploRA confocal spectrometer (Jobin Yvon, Horiba Gr, Palaiseau, France) with a 532 nm Nd:YAG laser light source. The instrument was calibrated against the Stokes Raman signal of pure Si at 520 cm^−1^ using a silicon wafer, and was equipped with a 600 lines/mm diffraction grating. A multichannel charge-coupled detector (1024 × 256 pixels) was used to collect spectra with a resolution of 3 cm^−1^. The spectra were scanned 10 times to ensure accuracy and improve the signal-to-noise ratio. A laser beam (2 mW) was focused on the sample surface to avoid damaging the WPAC.

XPS experiments were carried out on an RBD 147 upgraded PHI-5000C ESCA system equipped with a dual X-ray source, using the Mg Kα (1253.6 eV) anode and a hemispherical energy analyzer. The background pressure during the test was kept below 10^−6^ Pa, and the measurements were carried out at a pass energy of 93.90 eV. The samples were dried under vacuum before XPS analysis. The calibration of the binding energy of the spectra was performed with the C 1*s* peak at 284.6 eV. Data analysis and processing were performed using the XPSPeak 4.1 software with the Shirley-type background.

## 3. Results and Discussion

### 3.1. Cr Adsorption

**Figure 1:**
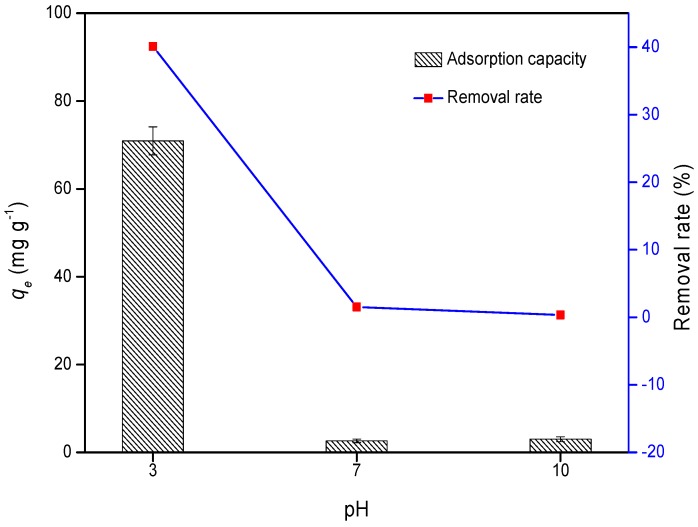
*q_e_* and the removal rate of Cr(VI) on wood-based powdered activated carbon (WPAC) at pH 3, 7, and 10.

The Cr(VI) adsorption capacity of WPAC was investigated at pH 3, 7, and 10. As can be seen from Figure 1, *q_e_* increased from 2.64 mg/g to 70.95 mg/g with decreasing pH from 7 to 3. The maximum adsorption was obtained at an initial pH of 3 with the removal of 40.04% of Cr(VI), whereas only 0.34% was removed at pH 10. Hence, Cr adsorption by WPAC was facilitated at low pH values. Previously, Shi [29] demonstrated that Cr(VI) existed as HCrO_4_^−^ (89%) and Cr_2_O_7_^2−^ (11%) at pH 3. As pH increased, Cr_2_O_4_^2−^ became the predominant form. Relevant studies showed that the degree of protonation of functional groups on activated carbon surface is enhanced under acidic conditions rather than alkaline conditions, leading to significantly strong electrostatic attraction between a positively charged activated carbon surface and chromate anions. Therefore, the adsorption capacity of the adsorbent was clearly pH-dependent. A similar trend was observed by Babel et al. [30], Selvi et al. [31], and Ihsanullah et al. [21], who proposed the reduction of Cr(VI) to Cr(III) under acidic conditions, with the concomitant oxidation of the carbon surface to form new oxygen-containing functional groups. These results can be explained by Equation (3). That is, the possible explanation for higher adsorption under acidic conditions is that Cr(VI) is oxidized to Cr(III). However, this mechanism was not confirmed by further investigations.

HCrO_4_^−^/Cr_2_O_7_^2−^ + R-H + H^+^ → Cr^3+^ + R-OH/R=O (3)

where R represents the activated carbon matrix.

### 3.2. SEM-EDS Analysis

**Figure 2:**
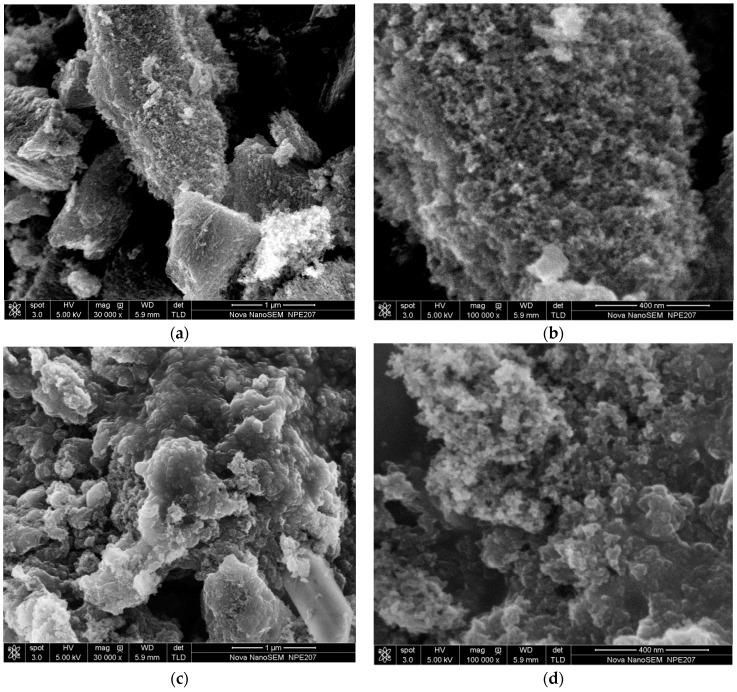
SEM images of WPAC ((**a**,**b**) before Cr(VI) adsorption and (**c**,**d**) after Cr(VI) adsorption, at pH 3. Scale bars: (**a**) 1 μm; (**b**) 400 nm; (**c**) 1 μm; and (**d**) 400 nm).

**Figure 3:**
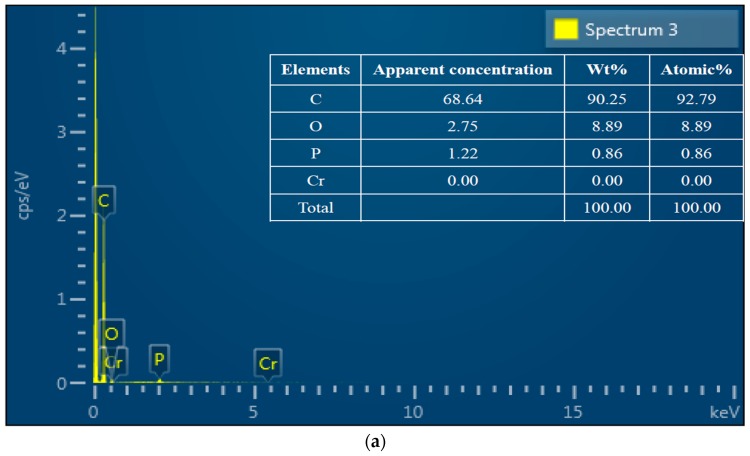

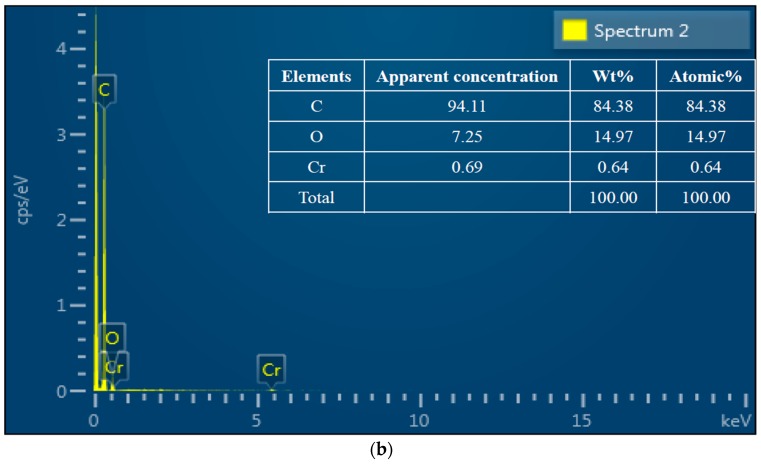
Energy dispersive spectroscopy (EDS) spectra of WPAC ((**a**,**b**) before and after Cr(VI) adsorption, respectively, at pH 3).

The morphology and porous structure of WPAC were characterized by SEM analysis before and after Cr(VI) adsorption. As shown in Figure 2a,b, the structure of virgin WPAC was irregular with uneven and rough surfaces containing macro- and micro-pores of various sizes and shapes, which supplied effective adsorption sites and spaces. After Cr(VI) adsorption, the WPAC surface showed an agglomerated morphology, and the pores were covered by Cr(VI), as can be seen from Figure 2c,d. Quantification of elements on WPAC was carried out using the SEM-EDS analysis software. As shown in the EDS spectra in Figure 3a,b, for virgin WPAC, the weight percentages of carbon, oxygen, and Cr(VI) were 90.25%, 8.89%, and 0%, respectively. After adsorption, the percentage of carbon decreased to 84.38%, whereas that of oxygen and Cr(VI) increased to 14.97% and 0.64%, respectively. The presence of Cr(VI) indicated that WPAC is an effective adsorbent for Cr(VI). Also, the increased amount of oxygen in the WPAC is attributed to the formation of oxygen-containing functional groups. Clearly, the oxygen/carbon (O/C) ratio in WPAC increased from 9.85% to 17.74% after Cr(VI) adsorption, which suggested that some graphitic carbon was oxidized with concomitant reduction from Cr(VI) to Cr(III) according to Equation (3). The oxidation process of functional groups on the adsorbent surface and their final forms of existence were verified by XPS and Raman spectroscopy.

### 3.3. Raman Spectroscopy

**Figure 4:**
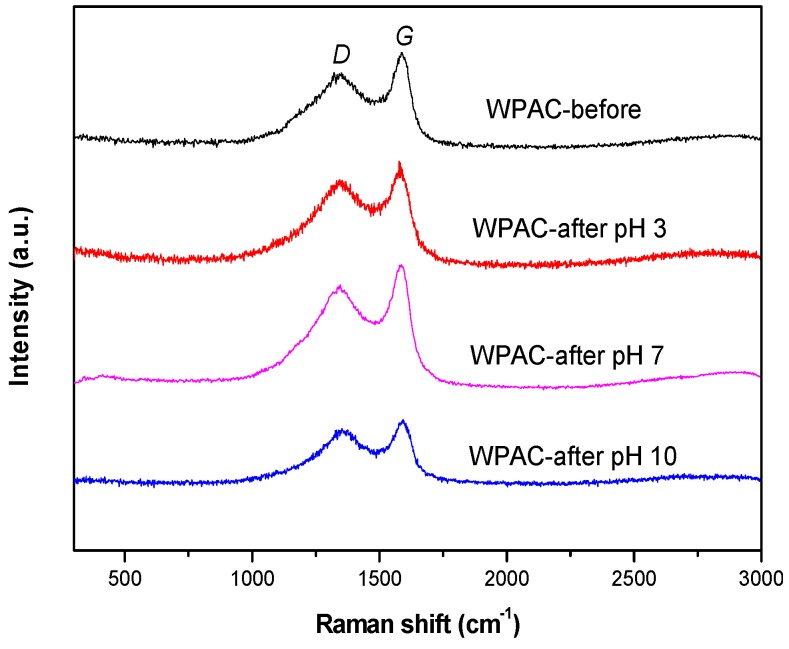
Raman spectra of WPAC before and after Cr(VI) adsorption at pH 3, 7, and 10.

Raman spectroscopy is a widely used, nondestructive method to study the ordered and disordered crystal structures of carbon materials [25], providing more detailed information on their crystallinity. Valuable data on the structure of carbon materials were collected in the spectral range of 300–3000 cm^−1^. As shown in Figure 4, all samples exhibited two characteristic peaks at 1360 and 1588 cm^−1^ denoted as the *D*-band and *G*-band, respectively, at pH 3, 7, and 10. The *G*-band arising from the stretching vibration of all *sp*^2^-bonded pairs indicates complete graphitization of the material [32], whereas the *D*-band is associated with the *sp*^3^ defect sites, indicating the presence of disorder [33]. The ratio of the integral intensities of the *G*- and *D*-bands (*I_D_*/*I_G_*) is usually used to measure the degree of defects in carbon materials [34]. The *G*- and *D*-band widths and *I_D_*/*I_G_* ratios are shown in Table 1. An *I_D_*/*I_G_* ratio greater than 1 was observed, which is typical of amorphous carbon materials and indicated a large number of defects in the structure. Under these conditions, the adsorption process was controlled by physisorption and prone to desorption. After Cr(VI) adsorption, the *I_D_*/*I_G_* ratio increased from 3.22 to 3.84 at pH 3, but decreased to 3.17 at pH 10. Under neutral conditions, although the *D*- and *G*-band values increased, no significant changes were observed for *I_D_*/*I_G_*. The R value was decreased with increasing pH value, which is to say that the disorder was more pronounced under acidic conditions than alkaline conditions. The result showed that the certain amount of structural disorder was generated by the preferential attack of oxygen species on the surface of the carbon [26]. The change in disorder was attributed to the change in the number of defects [35]. This result suggests that a chemical reaction occurred between the adsorbate and WPAC under acidic and basic conditions, which was consistent with the XPS results.

### 3.4. XPS Analysis

**Figure 5:**
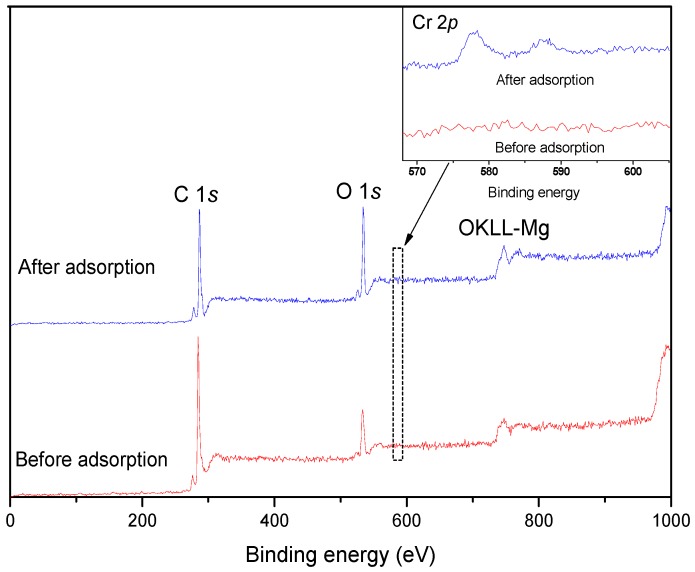
X-ray photoelectron spectroscopy (XPS) survey spectra of WPAC before and after Cr(VI) adsorption at pH 3.

The elemental composition and chemical oxidation state of the WPAC surface were analyzed by XPS. Distinct peaks of C (1*s*), O (1*s*), and Cr (2*p*) were observed, as shown in Figure 5, and Cr species were detected after Cr(VI) adsorption. The relative atomic concentrations of carbon, oxygen, and chromium are listed in Table 2. After adsorption, the O/C ratio increased from 0.24 to 0.32, suggesting an increase in oxygen-containing functional groups, which may be due to the reduction of Cr(VI). The results were in accordance with the SEM-EDS analysis.

**Figure 6:**
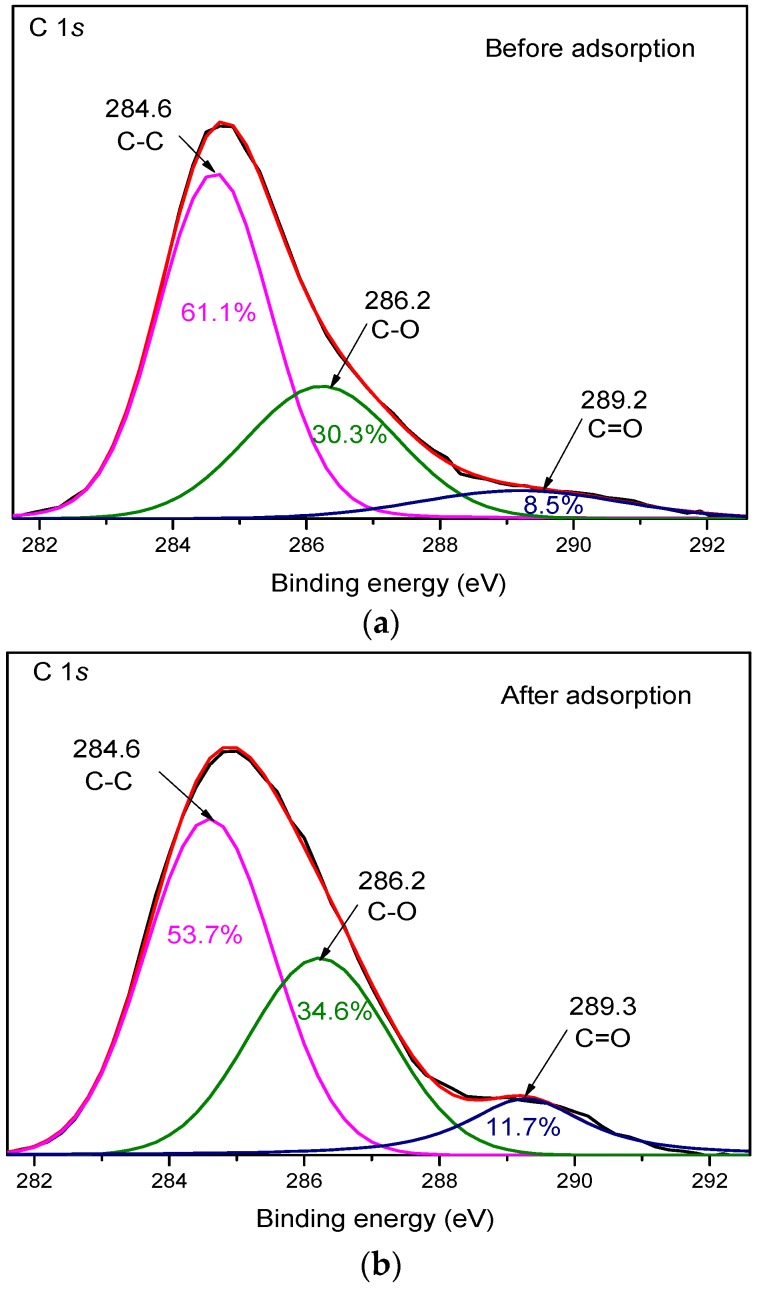
High-resolution XPS spectra of C 1*s* for WPAC ((**a**,**b**) before and after Cr(VI) adsorption, respectively, at pH 3).

To further confirm the increase in the O/C ratio on the WPAC surface, the typical high-resolution and curve fitting of C 1*s* spectra for Cr(VI) adsorption were recorded and are shown in Figure 6. The C 1*s* spectra were deconvoluted into three symmetric Lorentzian-Gaussian peaks, identified as the graphitic C-C bond at 284.6 eV (peak I), the C-O bond of hydroxyl or ether functional groups at 286.2 eV (peak II), and the C=O bond of carbonyl groups at 289.3 eV (peak III) [24,36,37]. According to the calculated peak areas (Table 3), the relative area of peak I for Cr(VI) adsorption decreased from 61.1% to 53.7%, whereas the relative area of peak II and peak III increased from 30.3% to 34.6% and from 8.5% to 11.7%, respectively. The increased amount of oxygen-containing functional groups on the surface of WPAC such as hydroxyl, carboxyl, and carbonyl provided more electrons for the reduction of Cr(VI) to Cr(III). This indicates that some graphitic carbons were oxidized during Cr(VI) adsorption. The possible oxidation reactions are shown in Equations (4) and (5):

(4) $$-C-H-e^{-}\overset{\mathrm{Cr}(\mathrm{VI})}{\to }-C-\mathrm{OH}$$

(5) $$-C-\mathrm{OH}-e^{-}\overset{\mathrm{Cr}(\mathrm{VI})}{\to }-C=O$$

**Figure 7:**
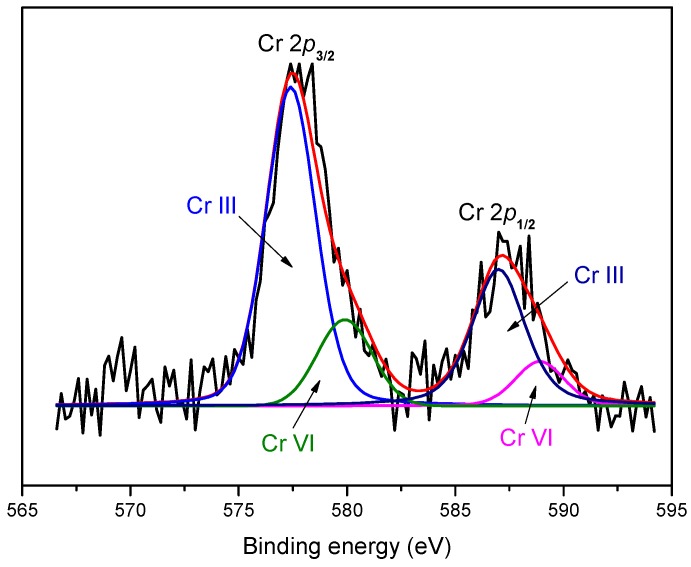
High-resolution XPS spectra of the 2*p*_1/2_ and 2*p*_3/2_ regions for Cr(VI)-containing WPAC.

High-resolution XPS spectra were measured to determine the valence states of the Cr species on the WPAC surface, and are shown in Figure 7. The optimum fitting was achieved by the deconvolution of the Cr 2*p* spectrum into four peaks (Table 4): the peaks at 577.4 eV and 586.9 eV were assigned to Cr(III) 2*p*_3/2_ and 2*p*_1/2_, respectively, whereas the other two peaks at 579.9 eV and 588.9 eV were attributed to Cr(VI) 2*p*_3/2_ and 2*p*_1/2_, respectively [38,39]. It is proposed that, after adsorption, Cr(III) and Cr(VI) coexisted on the surface of WPAC. As can be seen from the XPS spectra, about up to 78.8% of adsorbed Cr was in the trivalent state, indicating that, during adsorption, the majority of Cr(VI) was reduced to less toxic Cr(III) with the concomitant oxidation of C-H and C-OH. The removal mechanism of Cr(VI) is explained as follows: (1) interception of Cr(VI) species by the porous structure of WPAC; (2) binding of Cr(VI) anions to the positively charged surface of WPAC by electrostatic attraction; and (3) the majority of the adsorbed Cr(VI) anions are reduced to Cr(III) by the oxidation of some graphitic carbons, followed by adsorption and complexion with the oxygen-containing functional groups on WPAC.

## 4. Conclusions

SEM-EDS, Raman spectroscopy, and XPS were used to investigate the mechanism of Cr(VI) adsorption by WPAC. Raman spectra suggested a structural change of WPAC attributed to a change in the number of defects under acidic and basic conditions after Cr(VI) adsorption. This was confirmed by XPS and SEM-EDS analyses, which showed that the O/C ratio in WPAC increased significantly after adsorption. 

XPS showed that 78.8% of Cr adsorbed on the WPAC surface was in the trivalent state. The amount of oxygen-containing functional groups on the surface increased due to the oxidation of graphitic carbons to form C-OH and C=O groups, confirming the oxidation-reduction reaction between Cr(VI) and the adsorbent. These analytical techniques provided new insights in the Cr(VI) adsorption mechanism from a microscopic point of view.

Hence, Cr(VI) removal by carbonaceous materials was based on the reduction of Cr(VI) to Cr(III) followed by Cr(III) adsorption. Cr(VI) adsorption was highly dependent on the pH value, and the best result was obtained under acidic conditions (pH 3) because of the easier oxidation of C-H and C-OH to C-OH and C=O, respectively, accompanied by the reduction of Cr(VI) to Cr(III). Under acidic conditions, the carbon surface was positively charged due to protonation, leading to electrostatic attraction with negatively charged Cr(VI) anions (HCrO_4_^−^ and Cr_2_O_7_^2−^). Simultaneously, the ionization of Cr(VI) was facilitated at low pH [40]. Hence, the conversion of Cr(VI) to less toxic Cr(III) under acidic conditions by activated carbon is an effective way to remove Cr(VI) from aqueous solutions. 

## Figures and Tables

**Table 1:** *G*- and *D*-band widths and *I_D_*/*I_G_* ratios for WPAC before and after Cr(VI) adsorption at pH 3, 7, and 10.

Sample	*I_D_*/*I_G_*	*D*-Band Width (cm^−1^)	*G*-Band Width (cm^−1^)
WPAC-before	3.22	288.52	340.30
WPAC-after pH 10	3.17	209.63	224.80
WPAC-after pH 7	3.22	328.08	359.45
WPAC-after pH 3	3.84	327.44	310.65

**Table 2:** XPS analysis of elements on the WPAC surface.

Sample	C	O	Cr	O/C
Before adsorption	80.58%	18.43%	0	0.24
After adsorption	78.38%	20.54%	1.08%	0.32

**Table 3:** C 1*s* peak for WPAC before and after Cr(VI) adsorption at pH 3.

Sample	Parameters	C-C	C-O	C=O
Before adsorption	Peak position (eV)	284.6	286.2	289.2
	Peak area	55,087.02	27,323.53	7691.37
After adsorption	Peak position (eV)	284.6	286.2	289.3
	Peak area	35,748.62	23,006.97	7755.30

**Table 4:** Cr 2*p* peak for WPAC before and after Cr(VI) adsorption at pH 3.

Species	Parameters	Cr 2*p*_3/2_	Cr 2*p*_1/2_
Cr(III)	Peak position (eV)	577.4	586.9
	Peak area	5759.27	2879.63
Cr(VI)	Peak position (eV)	579.9	588.9
	Peak area	1549.87	774.93
